# Occult Leydig Cell Tumour Presenting as Bilateral Gynaecomastia. Case Report and Literature Review

**DOI:** 10.1100/tsw.2005.107

**Published:** 2005-11-08

**Authors:** A. B. Patel, L. Wilson, A. Rane

**Affiliations:** ^1^ Institute of Urology and Nephrology, University College London, UK; ^2^ East Surrey Hospital, Redhill, UK

**Keywords:** gynaecomastia, malignancies

## Abstract

Gynaecomastia is the most common benign breast disorder in men. Among the various causes, testicular malignancies are an uncommon, life-threatening condition requiring prompt diagnosis and treatment. The case of a 28-year-old man is discussed, who presented with a 6-month history of painful bilateral gynaecomastia with no abnormality on clinical or biochemical examination. The patient's symptoms spontaneously resolved within 4 weeks. He then represented 10 years later with similar symptoms and an associated secondary hypogonadism. Ultrasound imaging revealed a clinically occult, hypoechoic mass in the left testis (Leydig cell tumour on histology). Clinical and hormonal findings normalized following surgical excision. This report underlines the importance in clinical practice of ultrasonographic evaluation of the testis, in all patients with gynaecomastia, despite unremarkable findings on physical examination.

## INTRODUCTION

With an increasing incidence of testicular cancer over the last 30 years, public awareness campaigns have led to men seeking reassurance from urologists about a multitude of testicular symptoms. Clinical examination followed by high-resolution ultrasound scan can usually provide the reassurance needed. Rarely, men may present with endocrine manifestations (such as gynaecomastia) of a functioning testicular tumour. Even more rarely, the testicular tumour is occult, i.e., impalpable.

Leydig cell tumours (LCT) are rare testicular neoplasms that account for 1–3% of all testicular neoplasms[1,2]. They typically present in adults between the ages of 30–35 years as a testicular mass or with endocrine disturbance. A variety of hormonal manifestations such as male sexual precocity in children and gynaecomastia in adults are associated with testicular LCT due to secretion of estrogens and testosterone from the tumour cells[3]. Gynaecomastia can present long before evidence of LCT at clinical examination. We report a case in which bilateral gynaecomastia was induced by an occult testicular LCT. Though uncommon, similar cases have been reported in the literature and the aim of this report is to underline the importance of a thorough biochemical and physical examination in any patient presenting with unexplained gynaecomastia in order to exclude the possibility of an occult testicular tumour. The role of testicular ultrasound and selective venous sampling to detect a hormonally functioning occult testicular tumour is also discussed.

## CASE REPORT

A 28-year-old man originally presented to his primary care practitioner with a 6-month history of bilateral gynaecomastia. His relevant medical history was negative for hepatic, renal, or systemic disease, alcohol use, and medication. Endocrine investigation at that time (including prolactin, serum hCG, FSH, LH, and testosterone) proved normal and conservative management (outpatient follow up only – no interventional treatment) was followed. At 6-month follow-up, the gynaecomastia had resolved spontaneously and the patient was discharged from clinic.

He then represented 10 years later with recurrent bilateral grade II gynaecomastia. General examination revealed no other abnormality including clinically normal testes. Subsequent biochemical investigations revealed normal prolactin at 16 ng/ml (3–30), elevated 17-β estradiol at 2.6 nmol/l, low FSH at <1 mIU/ml (2.5–10.2), low LH at 1 mIU/ml (1.5–9.3), and low testosterone at 5.2 nmol/l (10.0–28.0). Thyroid and adrenal functions were normal, as were tumour markers CEA, αFP, and β-hCG. Testicular ultrasound (Fig. 1) revealed a hypoechoic 12-mm mass in the left testis. Subsequent staging computerised tomography of the thorax and abdomen proved normal. The patient then underwent a left radical orchidectomy; subsequent histology revealed a benign LCT with no evidence of malignancy. Following surgery, the LH, FSH, and testosterone levels normalised (15.3 nmol/l). Surprisingly, the prolactin level was elevated compared to baseline values, but remained at the upper limit of normal (24 ng/ml). A magnetic resonance imaging study was ordered as a precaution and revealed no evidence of a pituitary adenoma. However, by 3 months, this had resolved to baseline levels. The bilateral gynaecomastia resolved and the patient was discharged from follow-up endocrine clinic at 1 year (he continues to undergo regular urology surveillance).

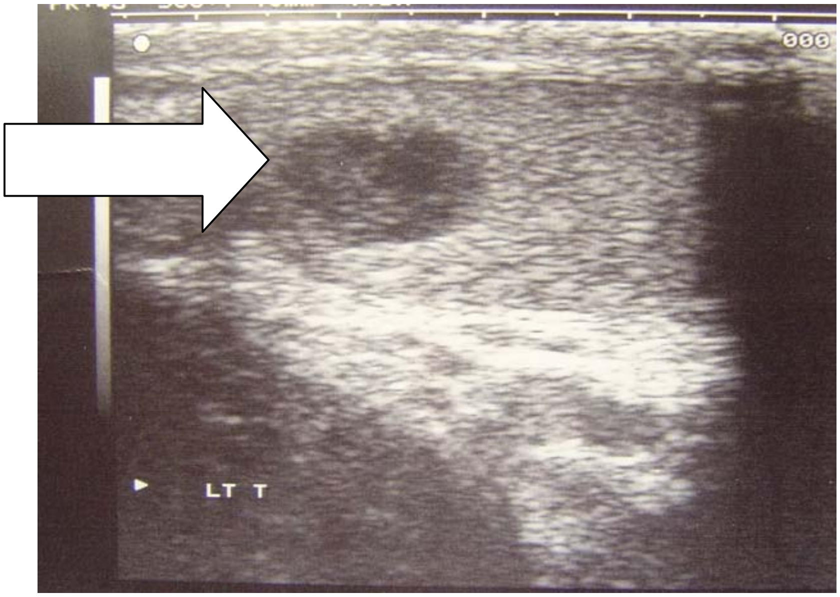

**FIGURE 1**. Left testicular USS showing a 12-mm hypoechoic tumour.

## DISCUSSION

The incidence of testicular cancer is rising. Over the last 25 years, age standardised rates in England and Wales have increased by 88%[4]. The vast majority of these tumours are palpable, the diagnosis then confirmed with ultrasonography. These cases pose little diagnostic difficulty. However, diagnosis can be delayed or missed when clinical presentation is atypical, such as with endocrinological disturbance associated with a normal testicular examination. Overall, gynaecomastia occurs in 15–25% of patients with LCT[5], which accounts for 3% of testicular neoplasms. This report underlines the importance of ultrasonographic evaluation of the testes whenever breast enlargement occurs in a healthy man, despite unremarkable findings on testicular examination.

LCT may occur in a patient of any age, but they have a bimodal peak incidence in the prepubertal age group and in patients between 20 and 50 years old. Histopathological features typically include a spherical and lobulated shape and are usually tan to brown in colour. Microscopically, sheets and nests of Leydig cells, usually with eosinophillic cytoplasm, are separated by fibrous septae, and in approximately 50% of cases, crystalloids of Rheinke may be seen as cigar-shaped intracytoplasmic inclusions[6,7].

The diagnosis of LCT can be very difficult. In children, clinical presentation nearly always manifests as isosexual precocious puberty[8]. In adults, about 20% of LCT present with an endocrine disturbance (either gynaecomastia or impotence) and as in this instance, the feminisation symptoms often precede the discovery of the testicular tumour by a long time. Typical hormonal LCT patterns include plasma estradiol levels >300 pmol/l on day 3 following hCG stimulation, reduced plasma testosterone, and normal plasma hCG and gonadotropin levels[9]. In this instance, the patient's hypogonadotropic hypogonadism is secondary to the negative feedback effect of elevated levels of estrogens on the hypothalamus and pituitary. In cases of normal estradiol levels and suspicion of LCT, the HCG test may be performed. An increased estradiol response to hCG testing has been described in patients with LCT and it depends on the increased production of estradiol[10] itself or increased aromatisation of testosterone[11]. Of the tumours, 10% are bilateral and 7–10% metastasise (the only true feature of malignant behaviour in these tumours)[12]. Patients with malignant LCT are likely to be older; the average age in this group is 67 years. Once metastases occur, the prognosis is uniformly poor since the disease is refractory to radiation or chemotherapy.

Gynaecomastia is the most common benign breast condition seen in men and is defined as a generalised enlargement of the male breast. It is common (40–65% of adult males) and may be unilateral or bilateral with the majority of cases reflecting an elevated circulating ratio of estrogens to androgens due to age, disease, drugs, or idiopathic factors[13]. Among the various causes of gynaecomastia, testicular malignancies are an uncommon and rarely a life-threatening condition, which requires prompt treatment. The pathophysiological mechanism is related to the reduced testosterone/17β-oestradiol ($E_2$) ratio, as a result of either increased production of $E_2$ or enhanced aromatise activity by the tumour itself[14]. For this reason, all patients presenting with gynaecomastia must have their testes carefully examined in addition to a full hormonal profile.

Review of the literature suggests that gynaecomastia preceding a readily palpable tumour is a recognised problem. There is a report of a case of progressive, bilateral tender gynaecomastia who underwent a bilateral subcutaneous mastectomy without regard for a possible occult testicular tumour. Following the mastectomy, metastatic testicular cancer was discovered; the patient had an overlooked scrotal mass and a very elevated βHCG[15].

Of particular interest in this case is that the initial endocrine disturbance had manifested a decade previously and biochemical investigation revealed no hormonal disturbance. Of note, no testicular ultrasound was performed at that time. Interestingly, the patient's symptoms spontaneously resolved before re-emerging 10 years later.

The objective of this case report is to alert physicians to the importance of use of testicular ultrasonography in patients with progressive gynaecomastia despite normal findings on testicular examination. The use of hormonal studies in addition to scrotal imaging is essential in the diagnosis of this tumour type and should be performed even in the absence of testicular anomalies on palpation.
